# Assessing pricing and affordability of HBV treatment in Asia–Pacific region: a barrier to elimination

**DOI:** 10.1007/s12072-024-10744-9

**Published:** 2025-02-03

**Authors:** Jing Chen, Jidong Jia, Hui Zhuang, Wenhong Zhang, Jin Mo Yang, Tawesak Tanwandee, Diana Payawal, Saeed Hamid, Shiv Kumar Sarin, Masao Omata, Guiqiang Wang, George Lau

**Affiliations:** 1 JC School of Public Health and Primary Care, Faculty of Medicine, Chinese University of Hong Kong, Hong Kong SAR, China; 2 Liver Research Center, Beijing Friendship Hospital, Capital Medical University, Beijing, China; 3 Department of Microbiology and Centre for Infectious Diseases, Peking University Health Science Centre, Beijing, China; 4 Department of Infectious Diseases, National Medical Center for Infectious Diseases, Shanghai Key Laboratory of Infectious Diseases and Biosafety Emergency Response, Huashan Hospital, Fudan University, Shanghai, 200040 China; 5 Department of Internal Medicine, St. Vincent’s Hospital, College of Medicine, The Catholic University of Korea, Seoul, 06591 Korea; 6 Division of Gastroenterology, Department of Medicine, Faculty of Medicine Siriraj Hospital, Mahidol University, Bangkok, Thailand; 7 Department of Medicine, Cardinal Santos Medical Center, Mandaluyong, Philippines; 8 Aga Khan University, Karachi, 74800 Pakistan; 9 Department of Hepatology, Institute of Liver and Biliary Sciences, New Delhi, India; 10 Yamanashi Hospitals (Central and Kita) Organization, 1-1-1 Fujimi, Kofu-Shi, Yamanashi, 400-8506 Japan; 11 Department of Infectious Disease, Center for Liver Disease, Peking University First Hospital, Beijing, China; 12 Department of Infectious Disease, Peking University International Hospital, Beijing, China; 13 Humanity and Health Clinical Trial Center, Humanity and Health Medical Group, 14F/21F,9 Queen’s Road Central, Central, Hong Kong SAR, China; 14 Zhongshan Hospital, Fudan University, Shanghai, China

**Keywords:** Hepatitis B virus (HBV), Asia–Pacific region, Affordability, Price, Health disparities

## Abstract

**Background:**

The Asia–Pacific (AP) region carries a substantial burden of HBV. Affordable HBV treatment is crucial to attain WHO’s elimination goal. This study assesses the pricing and affordability of HBV treatment in AP.

**Methods:**

A survey conducted among APASL members from 2 Aug to 30 Oct, 2023, gathered data on antiviral HBV drugs, treatment costs covering stages of chronic hepatitis B (CHB), compensated cirrhosis (CC), hepatocellular carcinoma (HCC), liver transplant, and monitoring expenses. Drug costs for TDF and ETV were compared to international reference price (TDF: $30, ETV: $36 per person per year), generating a median price ratio (MPR) where MPR < 1 indicated an acceptable local price. Affordability was evaluated by comparing yearly CHB treatment cost to yearly minimum wage in each country/area, all converted to 2023 US$.

**Results:**

ETV costs ranged from $42 per person per year in Pakistan to $2640 in Malaysia, while TDF costs varied from $12 in mainland China to $2446 in Hong Kong. Almost all MPR exceeded 1. Affordability of HBV treatment varied, with CHB patients in Australia paying 1.4% of minimum yearly wage to get 1 year CHB treatment, in contrast to Myanmar’s 78.6%. Affordability disparities were also evident for patients with CC, HCC, and liver-transplant needs, though monitoring costs were generally affordable.

**Conclusions:**

Despite patent expiration and availability of low-cost generics for TDF and ETV, HBV medication costs in Asia–Pacific region remain high. CHB treatment is generally unaffordable for patients, posing a significant barrier to HBV elimination in this endemic region.

## Introduction

Hepatitis B (HBV) is a major global health issue, ranking as the 10th leading cause of death and causing up to 1.2 million deaths annually [1]. Over 60% of these deaths occur in the Asia–Pacific (AP) region, where HBV contributes to 54.3% of global deaths from cirrhosis, 72.7% from hepatocellular carcinoma (HCC), and more than two-thirds of the global burden of acute viral hepatitis, leading to significant health and economic burdens [2].

Nucleos(t)ide analogues (NAs) such as entecavir (ETV), tenofovir disoproxil fumarate (TDF), tenofovir alafenamide (TAF), and pegylated interferon (pegIFN) are recommended as first-line therapies by guidelines from the Asian Pacific Association for the Study of the Liver (APASL) [3], the American Association for the Study of Liver Diseases (AASLD) [4], and the European Association for the Study of the Liver (EASL) [5]. These drugs are effective in viral suppression, reducing liver inflammation, improving histology, and enhancing outcomes in decompensated liver diseases [6–8]. However, their availability and affordability remain a significant issue, particularly in AP developing countries. Current data show that only 59% of AP countries have access to TDF and ETV as first-line chronic hepatitis B (CHB) treatment [9]. The potency, resistance profiles, and costs of these medications vary, influencing long-term treatment outcomes [10].

About 15–40% of CHB patients without proper management developed severe disease including cirrhosis, liver failure, and HCC [11]. In contrast, adverse events in patients treated according to guidelines and closely monitored are as low as 2% [12]. Early detection, treatment, and regular surveillance can prevent disease progression and potentially save healthcare costs.

The World Health Organization (WHO) has set an ambitious goal to eliminate HBV as a public health threat by 2030. Addressing HBV treatment affordability and accessibility is essential for achieving this goal. This study aims to assess the pricing and affordability of HBV treatment across the AP region by analyzing antiviral medication costs and evaluating treatment affordability to highlight disparities and barriers to eliminating HBV in this region.

## Materials and methods

### Study design

A cross-sectional survey was conducted among members of the Asia–Pacific Association for the Study of the Liver (APASL) Viral Elimination Task Force. A questionnaire was emailed to members from 18 countries/areas in the AP from 2 August to 30 October, 2023. The survey aimed to gather comprehensive data on the costs associated with HBV treatment, including drug costs, treatment costs for different stages of HBV, and surveillance costs.

### Data collection

Data were collected on the costs of two primary nucleoside (acid) drugs: Tenofovir Disoproxil Fumarate (TDF) and Entecavir (ETV). These two drugs are listed as essential medicines for hepatitis B by the WHO Model List of Essential Medicines (23rd list) [13] and are effective in managing HBV infection. Costs were reported in USD per month in the questionnaire. If costs were provided on a per-pill basis, they were multiplied by 30 to estimate the monthly cost. In addition, the questionnaire covered the costs associated with the treatment of CHB, compensated cirrhosis (CC), HCC, and liver transplant. These costs were asked to be reported in USD per year. Surveillance costs for routine liver tests, including alanine aminotransferase (ALT), HBV DNA, hepatitis B surface antigen (HBsAg) quantification, and non-invasive fibrosis tests (NIT), were gathered and reported in USD per test. If the above costs were reported in local currencies, those were converted to USD using the 2023 exchange rate.

### Cost analysis

#### Price

The median price ratio (MPR), representing the ratio of one medicine’s median unit price to the international reference price (IRP), was used for price evaluation. MPRs were calculated to express how much greater or less the median local medicine price was than the IRP. The WHO/HAI suggests using the International Medical Products Price Guide by Management Sciences for Health as the IRP. However, since the price for entecavir (ETV) was unavailable in this guide, we used an estimated minimum product cost of US$36 per person per year as the IRP for ETV [14]. For TDF, the Guide’s recommended prices were based on 2015 data when TDF was still patented, so we used an updated procurement price of US$2.5 per month per person, equating to US$30 per person per year as the IRP for TDF [15]. The median price for each medicine type in each country/area was calculated if the price was reported by at least three members in the same country/area. The lowest price was used if the price was reported in the form of range. An MPR of one or less is considered efficient procurement in the public sector, while below 2.5 is efficient for the private sector. Since our participating members mostly resided within the public sector, we used an MPR of 1 as the cut-off point for acceptable prices.

#### Affordability

According to the WHO/HAI definition, drugs intended to treat chronic and ongoing conditions are generally deemed affordable if their monthly therapeutic cost, based on a standardized treatment regimen, does not exceed the daily wages of the lowest-paid unskilled government workers (LPGW). Since our costs for CHB treatment in different stages were reported as yearly costs, we evaluated affordability by comparing the yearly cost of CHB treatment to the yearly minimum wage in each country/area. The average price in each country and region was calculated if the price was reported by at least three participating members in the same country/area. The lowest price was used if the price was reported in the form of range for each country/area. All figures were converted to 2023 US dollars for consistency.

## Results

### Participants

Overall, we received 27 valid responses from 17 countries/areas. The vast majority (96%) of the responses were from public hospitals.

### Price

**Fig. 1:**
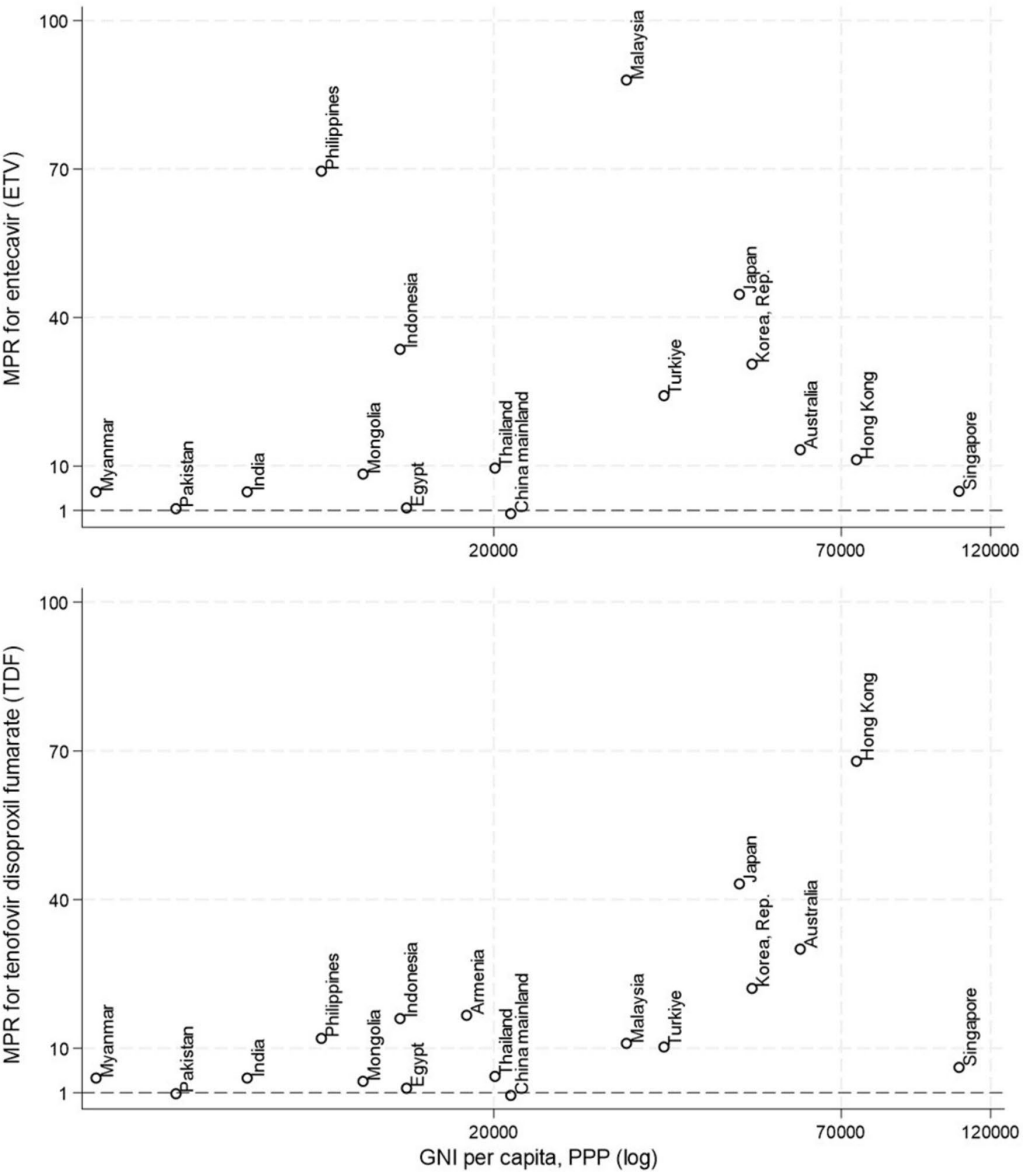
Median Price Ratio for Tenofovir Disoproxil Fumarate (TDF) and Entecavir (ETV) in surveyed countries/areas in Asia–Pacific region. Dot line represents MPR of 1 and 2. GNI per capita (PPP) is in log form

ETV costs per person per year ranged from $42 in Pakistan to $2640 in Malaysia, while TDF costs varied from $12 in mainland China to $2446 in Hong Kong (Table 1). The MPR for TDF and ETV varied widely across the AP region (Fig. 1). For TDF, the MPR ranged from as low as 0.5 in China to as high as 68.0 in Hong Kong. Countries such as Pakistan and Egypt had relatively low MPRs of 0.8 and 1.9, respectively. Conversely, countries such as Japan, Australia and Korea exhibited relatively high MPRs of 43.2, 30.0 and 22.1, respectively. Similarly, the MPR for ETV displayed a broad range, from 0.4 in China to 88.0 in Malaysia, with Egypt and Pakistan having lower MPRs of around 1.5. Philippines and Indonesia showed high MPRs of 69.6 and 33.6, respectively. MPR for TDF was marginally significantly correlated to gross national income (GNI) per capita (PPP) (correlation coefficients = 0.53, *p* = 0.03).

**Table 1:** Reported cost (USD) and median price ratio (MPR) of TDF and ETV per person per year

Country/region	ETV	MPR	TDF	MPR
Armenia	NR	NR	600.0	16.7
Australia	399.1	13.3	1081.3	30.0
China	17.6	0.4	12.0	0.5
Egypt, Arab Rep.	46.7	1.6	70.0	1.9
Hong Kong SAR, China	338.5	11.3	2446.2	67.9
India	144.0	4.8	144.0	4.0
Indonesia	1008.0	33.6	576.0	16.0
Japan	1341.1	44.7	1554.3	43.2
Korea, Rep.	918.0	30.6	795.6	22.1
Malaysia	2640.0	88.0	396.0	11.0
Mongolia	252.0	8.4	120.0	3.3
Myanmar	144.0	4.8	144.0	4.0
Pakistan	42.0	1.4	168.0	0.8
Philippines	2088.0	69.6	432.0	12.0
Singapore	147.6	4.9	221.3	6.1
Taiwan	1260.0	42.0	1380.0	38.3
Thailand	288.0	9.6	156.0	4.3
Turkiye	727.0	24.2	369.7	10.3

*NR* Not report, *TDF* Tenofovir Disoproxil Fumarate, *ETV* Entecavir, *MPR* median price ratio, ratio to the International Reference Price [ETV: US$36 per person per year, TDF: US$30 per person per year]

### Affordability

The affordability of HBV treatment varies significantly across different countries/areas in AP region, revealing significant disparities (Table 2). For example, in Australia, the yearly cost for treating CHB amounts to only 1.4% of the minimum yearly wage, indicating relatively high affordability. In contrast, in Myanmar the yearly CHB-treatment costs 78.6% of the minimum annual wage. This disparity persists across the more severe stages of the disease. In Thailand, CHB treatment requires 9.7% of the minimum annual wage, but the cost for HCC treatment is high at 1940.2%. Further, HCC treatment seems to be less affordable in more affluent countries. For example, in Pakistan HCC treatment takes up about 16% of the minimum yearly wage while in Hong Kong the figure is 522%.

**Table 2:** Affordability of annual HBV treatment (%)

Country/region	CHB	CC	HCC	Liver transplant	HCC surveillance
Armenia	26.88	26.88	NR	2240.14	10.75
Australia	1.39	3.31	220.58	1284.86	1.30
China mainland	8.12	21.56	121.68	1046.86	2.92
Egypt	20.89	25.53	63.45	2388.05	5.57
Hong Kong	39.20	65.34	522.69	2613.44	5.25
India	32.55	30.38	NR	5425.35	52.30
Indonesia	23.00	30.00	114.18	2778.12	11.84
Japan	NR	NR	NR	NR	NR
Korea, Rep.	5.09	5.09	260.34	477.83	2.25
Malaysia	8.04	8.04	NR	NR	37.94
Mongolia	27.44	43.90	87.80	2194.90	7.74
Myanmar	78.60	94.93	940.34	9469.70	48.53
Pakistan	7.22	7.22	15.83	2500.00	8.58
Philippines	3.09	3.09	231.91	7730.37	41.20
Singapore	NR	2.16	159.03	1657.45	8.25
Taiwan	34.82	38.99	278.52	1392.60	3.98
Thailand	9.70	19.40	1940.24	727.59	12.61
Turkiye	7.15	11.18	40.24	1117.67	2.02

Affordability was expressed as the percentage (%) of the annual treatment cost relative to the yearly minimum wage

*CHB* Chronic Hepatitis B, *CC* compensated cirrhosis, *HCC* hepatocellular carcinoma, *NR* Not report

Liver transplants remain unaffordable across the AP region, even in high-income countries, underscoring the severe economic burden of this life-saving procedure. For example, in South Korea, the cost of a liver-transplant amounts to 478% of the minimum annual wage, while in Myanmar, it soars to 9470%.

HCC surveillance, although generally more affordable, still exhibits significant variation across the region. In countries like Australia, South Korea, Turkiye and China, HCC surveillance costs less than 5% of the minimum annual wage. However, in countries like India and Myanmar, the cost is as high as 50%, reflecting considerable economic challenges for patients.

## Discussion

Pharmaceutical spending is projected to exceed $1.5 trillion globally by 2023, with significant increases across various regions [16]. In the AP region, despite the availability of generic medicines, rising drug costs pose a substantial challenge to healthcare systems for HBV. Our study highlights significant disparities in the pricing and affordability of CHB treatment across the AP region, underscoring the challenges in achieving equitable healthcare for CHB patients. These disparities have profound implications for the accessibility of treatment and the overall health outcomes for CHB patients, impacting progress toward the WHO’s HBV elimination goal.

WHO guidelines recommend TDF or entecavir (ETV) as the preferred first-line agents [17], with vanishingly low rates of virologic resistance [18]. Our study reveals a wide variation in the median price ratios (MPRs) for ETV and TDF, highlighting significant inequality in drug pricing within the AP region. For TDF, the MPR ranges from 0.5 in China to 68 in Hong Kong, while ETV prices vary from 0.4 in China to 88 in Malaysia. These disparities are indicative of differences in procurement efficiency, local pricing policies, and health system structures. For example, in China the central government adopted the “4 + 7” nationwide collective pharmaceutical procurement scheme, negotiating with drug manufacturers and wholesalers on the volume of medicines to be purchased [19]. Under this policy, 31 medicines including ETV and TDF were awarded contracts to manufacturers with the average price decreasing by 52% [20]. The procurement policy might explain the lowest MPR of TDF and ETV in China estimated from our study.

The availability of generic medicines also contributes to the low MPRs observed in countries such as India and Pakistan. In India, for example, the government has implemented policies to promote the production and use of generic drugs, including the establishment of Jan Aushadhi stores that provide affordable medicines to the public. The price for a standard pack of TDF and ETV from the online store is between 1 and 3 USD [21]. The Drug Regulatory Authority of Pakistan (DRAP) recently issued notifications directing all provincial governments with directives to prescribe medicines with their generic names in all public and private healthcare sector in the country [22]. It could contribute to a relatively lower MPRs of TDF and ETV in Pakistan and India than in other low-middle income countries in our study. With potent generic drugs, these initiatives could significantly reduce the cost of essential medicines like TDF and ETV, making them more accessible to the general population.

While there is ongoing debate about the incidence of HCC between entecavir (ETV) and TDF [23, 24], patient adherence emerges as a more critical factor influencing health outcomes [25]. Therefore, ensuring affordable drug costs, regardless of the specific medication, as financial barriers can significantly hinder adherence to treatment regimens. High costs reduce patients’ likelihood of maintaining adherence to prescribed therapies, potentially compromising their health and increasing the risk of adverse outcomes.

Our survey highlighted significant disparities in the affordability of treatments for HBV, including various stages such as CHB, CC, HCC, as well as associated procedures like liver transplantation and HCC surveillance across the AP region. The findings reveal a profound economic burden, particularly for low-income populations. In high-income countries like Australia and Korea, the cost of CHB treatment is relatively low, with modest financial burden of no more than 5% of the minimum annual wage. In contrast, less affluent countries such as Myanmar and India face much higher costs, making treatment of CHB less affordable. Interestingly, the affordability of treatments for more severe stages, such as HCC, is paradoxically lower in more affluent countries/areas. For example, in Hong Kong, HCC treatment costs exceed 500% of the minimum yearly wage. This may be attributed to the larger disparity between rich and poor in more affluent countries/areas, which exacerbates the financial burden on low-income populations. The Reimbursement policy coverage for CHB treatment might also play a significant role. For example, in some countries such as Japan, Australia and South Korea, the coverage for CHB treatment could be as high as 90% whereas the policy in India covers less than 5% of the rural population [26].

In countries with high treatment costs, such as those identified in our study, delays in diagnosis and inadequate management of CHB are more likely [27]. As a result, patients may experience progression to more severe liver conditions, including cirrhosis, liver failure, and HCC. This progression not only exacerbates the clinical burden on patients but also increases mortality rates, directly challenging the WHO’s target of reducing HBV-related deaths by 65% by 2030. Effective surveillance is crucial for early detection and management of liver disease progression, including the identification of HCC. It is vital that patients not only receive medications but also undergo necessary viral load checks and HCC surveillance; neglecting these aspects can undermine efforts to reduce disease burden. Despite successful HBV therapy leading to viral suppression, there remains a persistent risk of HCC [28]. This risk may be even higher among patients with poor adherence to treatment [29]. On-treatment monitoring is essential for ensuring patient adherence to medication and HCC surveillance. It also helps minimize the rates of loss to follow-up and self-discontinuation, which can lead to severe flares without timely management [30]. Therefore, the accessibility and affordability of HCC surveillance is extremely important. Our survey indicates that HCC surveillance is generally affordable in the AP region, which boosts our confidence in efforts to reduce the incidence of HCC and other severe complications associated with chronic HBV infection.

### Policy implications and recommendations

To address these disparities, several policy recommendations can be considered. First, regional cooperation can significantly enhance the procurement efficiency and reduce drug prices through collective bargaining. Countries in the AP region can benefit from coordinated ordering, pooled procurement, and the prequalification of products to address regulatory bottlenecks [31]. Collaborative efforts like the WHO’s Global Drug Facility for tuberculosis have successfully reduced drug prices and improved access, illustrating the potential benefits of similar initiatives for HBV treatment [32, 33].

Second, implementing universal health coverage (UHC) to ensure equitable access to essential HBV treatments, can achieve elimination of viral hepatitis [34]. UHC policies should include financial protection mechanisms to prevent out-of-pocket expenses from becoming a barrier to care. This approach is vital for low- and middle-income countries where the cost of HBV treatment can be relatively high. Financial protection mechanisms could encompass the full scope of CHB management, including diagnosis, treatment, regular surveillance, and government-funded healthcare services. These measures would ensure that patients, particularly those from low-income backgrounds, can access continuous care, facilitating timely diagnosis and effective disease management. This approach would ultimately reduce the long-term burden of CHB-related complications such as cirrhosis, liver failure, and HCC. By incorporating these considerations into UHC frameworks, we can prevent financial barriers from undermining the benefits of HBV treatment and make efforts toward achieving the viral hepatitis elimination.

Third, investing in healthcare infrastructure is crucial for the effective delivery of HBV care. Enhanced infrastructure could include higher coverage of vaccination, better diagnostic facilities, improved supply chains for medication, and advanced treatment centers [35]. Such investments can lead to early detection and timely management of CHB, thereby reducing the disease burden and yielding substantial returns [36]. A critical component of infrastructure in eliminating HBV is expanding neonatal vaccination programs, as the birth dose is highly effective and cost-efficient in preventing mother-to-child transmission, particularly in endemic regions. It has significantly reduced new infections [37] and liver cancer [38, 39], even eliminating liver cancer in vaccinated children in some areas [40]. However, birth-dose coverage remains suboptimal, with rates below 80% in the Western Pacific and under 60% in Southeast Asia [41]. Increasing neonatal vaccination coverage is essential not only for reducing new infections but also for minimizing the long-term costs of managing chronic infections and liver-related complications. Without addressing this key prevention strategy, efforts to eliminate HBV through treatment alone will face significant challenges.

Last but not the least, encouraging the use of generic medicines can substantially reduce the cost of anti-viral drugs which provide access to affordable and essential medicines in low and middle-income countries. Policies that support the local production of generic drugs, streamline regulatory approval processes, and promote the use of generics in healthcare systems can lead to significant cost savings.

### Limitations

This study has several limitations. First, the use of convenience sampling may not adequately represent the broader population, potentially introducing bias. The geographic coverage might not capture local variations within countries, affecting the overall findings. In addition, most data were sourced from the public sector, which may not reflect private sector pricing and availability, thus skewing the results. Although the data from the public sector were likely extracted from hospital clinical management systems, potential reporting errors remain a concern. The fact that most locations had only one response introduces reporting bias and limits generalizability. Furthermore, the analysis did not differentiate between costs for originator brands and generics, nor between costs covered by healthcare insurance and out-of-pocket expenses, obscuring variations in pricing structures. However, regardless of who bears the cost, the high costs associated with CHB treatment especially in low-income countries identified from current study pose a significant barrier and may delay progress toward the goal of viral elimination. Future surveys will be conducted to address these concerns. Variability in procurement practices and local pricing policies also complicates the comparability of price data across regions.

## Conclusion

Our survey identified significant disparities in drug pricing and treatment affordability for HBV across the Asia–Pacific region, both between high- and low-income countries and within high-income countries among different income groups. These disparities highlight the pressing need for coordinated policy efforts to improve access to affordable care. Addressing these challenges is vital for achieving the WHO’s goal of HBV elimination and for improving the health outcomes of millions of CHB patients in the region.

## Data Availability

All data will be shared upon request to the corresponding author.
